# Acid Reflux: A Rare Adverse Effect of Duloxetine

**DOI:** 10.7759/cureus.42327

**Published:** 2023-07-23

**Authors:** Nital Vaghela, Naresh Jadav, Shubha Kollampare, Pinki Patel, Meera Oza

**Affiliations:** 1 Internal Medicine, Government Medical College, Surat, Surat, IND; 2 Internal Medicine, New Civil Hospital, Surat, IND; 3 Rheumatology, Hospital Corporation of America (HCA) Florida Orange Park Hospital, Orange Park, USA; 4 Rheumatology, Arthritis and Osteoporosis Treatment Center, Jacksonville, USA; 5 Rheumatology, Hospital Corporation of America (HCA) Florida Orange Park Medical Center, Orange Park, USA

**Keywords:** multimorbidity, duloxetine, snri, rare adverse event, heart burn

## Abstract

Duloxetine is a medication that belongs to the serotonin and norepinephrine reuptake inhibitor (SNRI) class of drugs and is commonly used to treat various conditions, such as depression, generalized anxiety disorder, neuropathic pain, fibromyalgia, and chronic musculoskeletal pain. While side effects, such as headaches, constipation, dry mouth, dizziness, and blurred vision, are commonly associated with duloxetine, we present a case of a 59-year-old woman who experienced a rare adverse event of acid reflux while taking a 30 mg dose of duloxetine for fibromyalgia. According to the available literature, this adverse event has been reported in only 1.38% of the population.

## Introduction

Adverse drug reactions (ADRs) continue to pose a challenge in modern medicine, particularly considering the evolving complexity of therapies, the aging population, and the increasing prevalence of multimorbidity [1]. An ADR is defined as a medication-induced, noticeably harmful, or unpleasant response. Such reactions often signal potential risks associated with future treatments and necessitate preventive measures, specific treatments, adjustments in dosing schedules, or discontinuation of a product [2]. Duloxetine, a member of the serotonin and norepinephrine reuptake inhibitor (SNRI) family of drugs, is utilized for the treatment of major depressive disorder (MDD), fibromyalgia, generalized anxiety disorder, chronic musculoskeletal pain, and diabetic peripheral neuropathy. In addition, it is used off-label for conditions, such as stress incontinence and peripheral neuropathy resulting from chemotherapy. Duloxetine shares similar side effects with traditional selective serotonin reuptake inhibitors (SSRIs), with nausea being the most common adverse effect and often resolving upon discontinuation. Other typical side effects include constipation, insomnia, hypersomnia, dizziness, weakness, sleepiness, sedation, exhaustion, diarrhea, headaches, and xerostomia [3]. While certain antidepressants, such as trazodone, citalopram, fluoxetine, mirtazapine, or fluvoxamine, have shown a protective effect on gastrointestinal symptoms, the results are occasionally inconclusive. This case study focuses on an infrequent side effect of duloxetine and aims to raise physician awareness regarding its consideration and relevant variables when prescribing duloxetine to older patients with gastroesophageal reflux disease (GERD).

## Case presentation

A 59-year-old elderly female with a known history of seronegative inflammatory polyarthropathy had a documented allergy to sulfur, prompting the initiation of hydroxychloroquine 400 mg once daily. Hydroxychloroquine was subsequently discontinued due to headaches. The patient's medical history includes hyperthyroidism, osteoporosis, and peptic ulcer disease for which she used to be on proton pump inhibitors (PPIs). She has never smoked and does not consume alcohol. Her mother has a family history of severe arthritis. Duloxetine 30 mg daily was added for fibromyalgia in 2019, resulting in an improvement in her symptoms. In addition to the medications, she was receiving denosumab 60 mg subcutaneously every six months for osteoporosis, along with daily calcium supplements of 1200 mg and vitamin D3 1000 IU. After five years of initiating duloxetine, the patient reported worsening GERD with complaints of heartburn. Gastroenterology consultation led to a diagnostic endoscopy of the upper and lower gastrointestinal tracts, which revealed gastritis. A test for *Helicobacter pylori* was conducted, and the results came back negative. PPIs were initiated and continued for a period of six weeks but did not alleviate her symptoms. The patient was subsequently referred to cardiology to evaluate for cardiac etiology masquerading as GERD-like symptoms. The diagnostic cardiac workup, including electrocardiogram and echocardiogram, yielded negative results. The patient was then advised to taper off duloxetine and followed up for eight weeks, subsequently improving her heartburn symptoms. Ultimately, she discontinued duloxetine and experienced complete relief from acid reflux, also noted on repeat endoscopy.

## Discussion

Duloxetine, an SNRI, exerts analgesic effects by enhancing 5-hydroxytryptamine (5-HT) and norepinephrine (NE) activities in the central nervous system [4]. Gastrointestinal adverse effects associated with the drug have long been recognized as significant and challenging problems impacting patient diagnosis and care. Duloxetine can cause several gastrointestinal adverse effects, with nausea, constipation, diarrhea, reduced appetite, dry mouth, and vomiting being the most frequently reported thus far [5]. Other very rare adverse events include duloxetine-induced tachycardia, hyponatremia, and self-limited kleptomania symptoms [6-8]. This paper presents a case of GERD-like symptoms associated with the use of duloxetine, which represents an extremely uncommon adverse event. A phase IV clinical study conducted by eHealthMe involving 186,349 individuals who reported side effects while taking medications containing duloxetine hydrochloride found that GERD was observed in individuals, particularly females aged 60 and above, who had been taking the medication for an extended period of time. Among the participants, 2,580 individuals (1.38%) reported GERD, with a higher incidence in females (84.38%) compared to males (15.62%) [9]. A more comprehensive study is warranted to elucidate this side effect further, as its mechanism is multifactorial and not yet fully understood.

## Conclusions

In our case study, duloxetine has been identified as a potential cause of heartburn, which is considered one of its rare adverse events. A key finding from our research is that the patient's heartburn improved and symptoms returned to normal after discontinuing this medication. This presentation may have been influenced by the patient's prolonged use of duloxetine over five years, suggesting a potential time-dependent effect. Although this rare adverse effect of duloxetine has been documented in the literature, it remains generally unknown among medical professionals. Therefore, the conclusion of this research contributes to the existing body of medical literature by describing an infrequent and lesser-known side effect of duloxetine. This side effect should be considered before prescribing duloxetine to individuals with pre-existing heartburn.

## References

[1] Coleman JJ, Pontefract SK (2016). Adverse drug reactions. Clin Med (Lond).

[2] Aronson JK, Ferner RE (2005). Clarification of terminology in drug safety. Drug Saf.

[3] Wernicke JF, Gahimer J, Yalcin I, Wulster-Radcliffe M, Viktrup L (2005). Safety and adverse event profile of duloxetine. Expert Opin Drug Saf.

[4] Woolf CJ (2004). Pain: moving from symptom control toward mechanism-specific pharmacologic management. Ann Intern Med.

[5] Rodrigues-Amorim D, Olivares JM, Spuch C, Rivera-Baltanás T (2020). A systematic review of efficacy, safety, and tolerability of duloxetine. Front Psychiatry.

[6] Yalçınkaya B, Tüten B, Yazar B, Demirel K, Ocak H (2023). Duloxetine induced tachycardia: a rare side effect. Pain Pract.

[7] Baig MU, Madden K, Moody K (2022). Hyponatremia with antidepressant: a rare side effect from duloxetine in a child with acute leukemia. J Palliat Med.

[8] Miller CW, Gallagher KE (2016). Self-limited kleptomania symptoms as a side effect of duloxetine. Case Rep Psychiatry.

[9] (2023). Gastroesophageal reflux disease and drugs of ingredients of duloxetine hydrochloride - a phase IV clinical study of FDA data. https://www.ehealthme.com/ds/cymbalta/gastroesophageal-reflux-disease/.

